# Chilblains

**Published:** 1899-02

**Authors:** 


CHILBLAINS.—C. Binz (*Fortschritte der Medicin*) thinks that only chemicals capable of penetrating the epidermis can be expected to have any effect upon chilblains. To these belong chlorine in the form of chlorinated lime. He has found that one part of this, mixed with nine parts of paraffin ointment, rubbed into the inflamed parts for five minutes every night, will cause the pain and swelling to disappear in the course of a week. After each inunction the foot is covered with a very thick bandage. It is important that the ointment should have a strong odor of chlorine, and he points out that the chlorinated lime of shops has generally parted with its free chlorine. Another point of importance is that the drug should be mixed only with paraffin ointment; for Binz has found that, when mixed with lard and especially with lanolin it gives up its chlorine too quickly. The ointment is useful only so long as it gives out a decided smell of chlorine.—*Indian Lancet.*

